# Pole Dancing-Specific Muscle Strength: Development and Reliability of a Novel Assessment Protocol

**DOI:** 10.3390/mps7030044

**Published:** 2024-05-18

**Authors:** Despoina Ignatoglou, Achilleas Paliouras, Eleftherios Paraskevopoulos, Nikolaos Strimpakos, Paraskevi Bilika, Maria Papandreou, Eleni Kapreli

**Affiliations:** 1 Clinical Exercise Physiology and Rehabilitation Research Laboratory, Department of Physiotherapy, School of Health Sciences, University of Thessaly, 35132 Lamia, Greece; dignatoglou@uth.gr (D.I.); apaliouras@uth.gr (A.P.); pbilika@uth.gr (P.B.); 2 Biomechanics Laboratory, Department of Physiotherapy, School of Health Sciences, University of Peloponnese, 23100 Sparta, Greece; elparaskevop@uniwa.gr; 3 Laboratory of Advanced Physiotherapy, Department of Physiotherapy, University of West Attica, 12243 Athens, Greece; mpapand@uniwa.gr; 4 Health Assessment and Quality of Life Research Laboratory, Department of Physiotherapy, School of Health Sciences, University of Thessaly, 35132 Lamia, Greece; nikstrimp@uth.gr

**Keywords:** pole dancing, Activ5, isometric strength, sport-specific

## Abstract

Background: Pole dancing is a physically demanding sport that combines dance and acrobatic movements on a vertical pole. Despite its highly growing popularity, there is currently limited research in the field. The aim of this study was to create and evaluate a strength assessment protocol for athletes in pole dancing, with a specific focus on functional positions on the pole. Methods: Thirty-two female pole dancing athletes participated in this study. Maximal voluntary isometric contractions (MVIC) were measured at three different sport-specific positions on the pole (shoulder abduction and adduction, and hip adduction), on two separate days (test and re-test) with a five to seven day interval between them. A hand-held dynamometer (Activ5- Activbody) stabilized on the pole was used for this study. Results: The intra-session reliability was good to excellent for all sports-specific positions and for both sides of the body, across all different movements (ICC = 0.837–0.960, SEM = 5.02 Kg–2.24 Kg, and SDD = 27.46%–14.92%). Slightly better results were found regarding inter-session reliability (ICC = 0.927–0.970, SEM = 3.72 Kg–1.97 Kg, and SDD = 22.86%–15.19%). There was not a statistically significant difference between the MVICs between the left and right or dominant and non-dominant side in shoulder abduction (*p* = 0.105) and hip adduction (*p* = 0.282), in contrast to shoulder adduction (*p* = 0.00). Conclusion: The strength assessment protocol developed in the current study has proven to be a reliable and functional tool, with the potential for utilization in clinical practice as part of objective strength testing. Further studies are needed in order to expand the protocol to other muscle groups and positions and to generalize the results in all pole dancing populations such as male athletes.

## 1. Introduction

The sport of pole dancing (PD) has undergone significant growth and popularity, particularly among amateur athletes, in recent years. With the assistance of organizations such as the International Pole Sports Federation (IPSF), PD has now evolved into an international sport with rigorous training regimens, complex scoring systems, and international championships [1]. It is a very demanding sport that combines acrobatic gymnastics and dance on and around the pole whereas it is considered to have developed from a combination of Western and Eastern practices, the latter originating from the Chinese and Indian traditions [1,2]. Despite the rapid growth of PD and the substantial number of individuals participating in it professionally or recreationally, there remains a limited amount of available research on PD, primarily in recent years [1,3,4].

**Figure 1:**
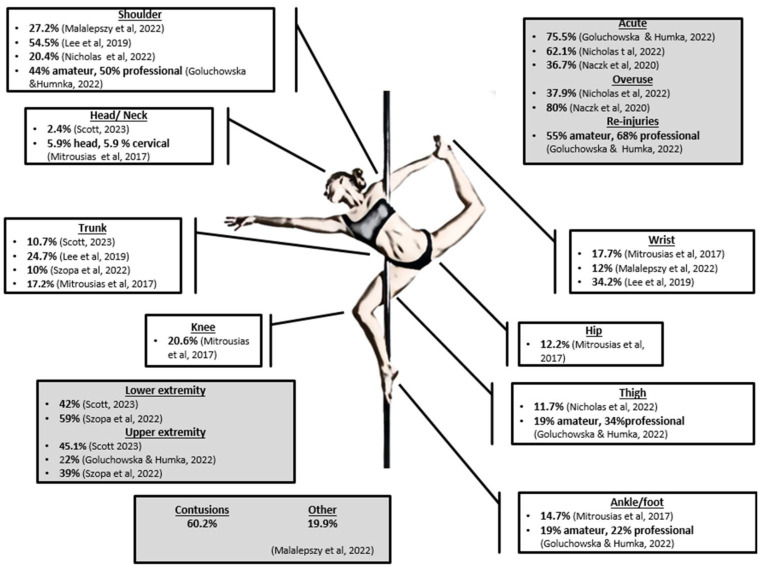
Epidemiological data of injuries in PD athletes [2,4,5,6,7,8,9,10].

Epidemiological studies on PD indicate that shoulder and wrist joints are the most frequently injured joints, while injuries to the pole itself typically involve sprains, bruises, and friction problems [2,5,6,7]. Professionals seem to sustain more injuries than amateurs, and they are more susceptible to re-injuries [6]. There are several factors that seem to contribute to injuries, such as age, height, training frequency, and duration of practice, while pole-specific training hours were associated with a higher injury rate [7,8]. Moreover, particular figures and positions, such as the handspring, twines, and carousel, have been identified as being associated with the highest incidence of problems [4]. In general, non-contact injuries are the most common (57.3%), with repeated shoulder rotations and postures involving front splitting being particularly prevalent [2]. Figure 1 presents epidemiological data on injuries derived from previous studies.

Strength assessment is an important clinical parameter, especially for athletes, providing values for training, evaluating injury risk, or determining return-to-play criteria [11]. Maximal voluntary isometric contractions (MVIC) testing is a type of physical assessment commonly used in athletes to evaluate muscle strength, endurance, and overall performance. It involves holding a muscle contraction without joint movement, providing a measurable and accurate measure of an individual’s muscular strength. The primary purpose of MVIC testing in athletes is to identify any muscular imbalances or weaknesses that may hinder their performance [12]. By measuring MVIC strength, coaches, trainers, and sports physiotherapists can obtain valuable insights into athletes’ strength profiles and pinpoint areas requiring improvement [12]. MVIC testing is especially applicable for athletes with a training routine and performance that includes isometric contractions such as in PD [11]. Dynamometers are the tools that are commonly used for MVIC strength assessment. In daily clinical practice, hand-held dynamometers are easy to use, affordable, and need limited training for the examiner without lacking reliability (ICC range = 0.89–0.97) [13].

In PD athletes, research has predominantly focused on hand grip strength, despite the involvement of various muscles in maneuvers and positions such as those of the shoulder and hip that are prevalent to injuries (Figure 1). Studies have focused on the positive correlation between the years of experience and the hand grip strength for both hands [1,3] or on the differences between dominant and non-dominant hands [1]. It has also been observed that PD athletes have significantly greater hand grip strength comparing with other female athletes from sports like weightlifting, volleyball, and swimming [1] or when compared with untrained females [4].

As a general rule, MVIC assessments provide force-time characteristics in specific locations, which may be obscured by length- and velocity-tension relationships [12]. As a result, sport scientists and practitioners must take into account the athlete’s particular sport when using MVIC testing. Essentially, the athletes must be evaluated in a position relevant to their sport’s biomechanical characteristics and needs. In this regard, there is a current tendency to develop protocols specifically designed for sports rather than using the same protocol across all sports. A study conducted with rock climbers resulted in the development of a valid sport-specific test battery designed to measure strength based on positions and movements commonly used in climbing. For example, finger hang and band-arm hang tests were developed [14]. Similarly, another study assessed the validity and reliability of a method for assessing sports rock climber’s MVIC finger strength using finger hang position, since this is the main position in which climbers use their fingers for climbing [15]. Further research conducted with ballet dancers involved the use of a dynamometer with an external stabilizer to assess the performance of multijointed lower extremity muscles during three common dance movements. This protocol was found to be reliable [16].

There seems to be no sport-specific strength assessment protocol for PD athletes in the literature. Therefore, the aim of this study was to develop and assess the intra-session (test) and inter-session (re-test) reliability of MVIC assessment of the three most used sport-specific positions on the pole, using a hand-held dynamometer. The secondary aims were to provide preliminary data for the MVIC strength of shoulder adduction, shoulder abduction, and hip adduction and the differences between the dominant and non-dominant side. It was hypothesized that (1) the MVIC strength assessment would be repeatable in all three positions, (2) MVIC strength values would be influenced by limb laterality and muscle group.

## 2. Materials and Methods

### 2.1. Sample

A sample of 32 female PD athletes were recruited from dance schools in the greater area of Thessaloniki and Halkidiki, Greece (Table 1). Subjects with pain, injury, history of surgeries, or skin disease of the body region as well as congenital problems (e.g., dysplasia, neurological deficits, etc.,) were excluded from the study. Additionally, participants were excluded if they were menstruating on either of the two measurement days. The study was approved by the Ethics Committee of the Physiotherapy Department of the University of Thessaly (645/09-09-2021) and performed according to the Declaration of Helsinki. The participants gave verbal and written informed consent to participate in the study.

**Figure 2:**
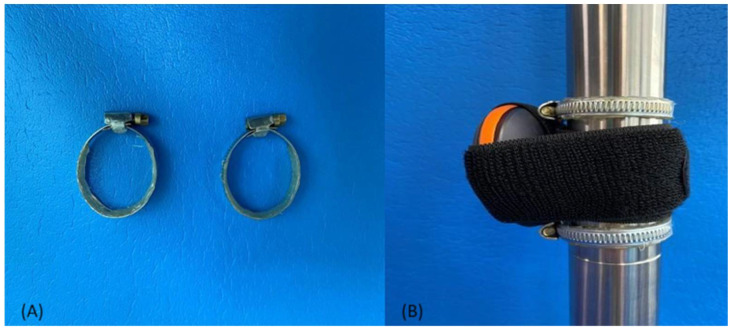
(**A**) Silicone coated clamps and (**B**) pole mounting method.

MVIC was measured with the Activ5 hand-held dynamometer of the Activbody Company (California Proposition 65). By utilizing a Bluetooth-enabled compressive load sensor, the device measures muscle strength, which is displayed via an application on the user’s smartphone. The Activ5 dynamometer has demonstrated excellent reliability (ICC = 0.999) in a test-retest situation with an external compression tool applied (Instron ElectroPuls E10000 (universal testing machine UTM)), along with high validity, particularly in low-load examinations as evidenced by agreement with a gold standard (ICC ≥ 0.971). These findings reinforce its suitability for use in the current study [17]. It has a measuring range from 0.0 to 90.0 kg with an accuracy of ±0.635 kg + 5% of the applied force. The dynamometer was fixed on the pole, secured from both the top and bottom ends with screwed metal clamps, while at the same time it was tied with a strap. Both the strap and the clamps were lined with silicone on the inside to prevent slipping of the dynamometer in any direction (Figure 2). The pole used for measurement was the Lupit Pole Classic Static/Spinning, chrome, with a diameter of 42.5 mm and was locked in its static mode.

The dominance of the lower limbs was assessed using the Greek version of the Waterloo Footedness Questionnaire- Revised (WFQ-R) [18], whereas the dominance of the upper limbs was based on the writing preference. WFQ-R is a 10-item questionnaire that assesses foot preference for manipulating objects (WFQ-Rm score) and for providing postural support during an activity (WFQ-Rs score). The items 1, 3, 5, 7, and 9 are intended to measure the dominant foot’s ability to manipulate objects, while the items 2, 4, 6, 8, and 10 are intended to measure the dominant foot’s ability to provide postural support. Responses of (a) left-always, (b) left-usually, (c) equal, (d) right-usually, (e) right-always are scored on a scale from ±2 to 2, giving a range of scores from −20 for the absolute left-footed to +20 for the absolute right-footed. Participants with scores between −7 and −20 can be categorized as left-footed, those with scores between −6 and +6 as mixed-footed, and those with scores between +7 and +20 as right-footed, according to Grouios et al. [19].

### 2.2. Procedure

The measurements took place in the gym of a physiotherapy clinic. The main examiner (I.D.) conducted the measurement protocol, while an assistant examiner was responsible for recording each measurement result (using a blind process). A pilot study was conducted with two examiners before the real measurements to identify any issues related to the measurement process or equipment usage.

The subjects filled out the consent form and the questionnaire (WFQ-R) at the beginning of the procedure. Their height and weight were measured at that time on an electronic scale and a measuring tape on the wall. Both measurements were conducted with the participants barefooted. The body mass index (BMI) was calculated and recorded. For the randomization of the positions and laterality, participants chose from two categories of closed opaque envelopes. One pack of envelopes included the positions (shoulder abduction, shoulder adduction, and hip adduction) and the participant was asked to place them randomly in an order, which was then followed by repeating the same procedure for the envelope of lateralization (left, right). Subjects were dressed with proper clothes for the procedure. Initially, the participant laid on an examination bed and the anterior superior iliac spine (ASIS) was marked. Then the participant sat on the edge of the examination bed and while adopting a relaxed arm position by resting it next to the body, the bony areas of the acromion was marked.

After completing the somatometric measurements, subjects approached the pole to start the force measurement procedure. The assessment began with the randomly selected position and extremity (left or right). Three different positions, two for the shoulder joint and one for the hip joint, were chosen for the strength assessment. Before measuring each position, subjects were provided with both oral instructions and a photograph to further explain the position on the pole.

**Figure 3:**
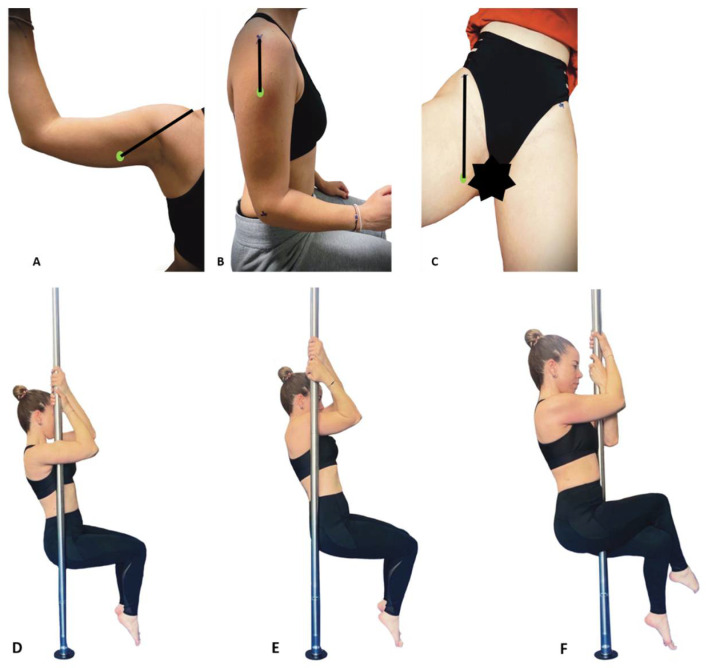
Centre of pressures (green spot) and distances (black lines) from (**A**) acromion for shoulder adduction, (**B**) acromion for shoulder abduction, (**C**) anterior superior iliac spine for hip adduction, (**D**) position for shoulder adduction, (**E**) position for shoulder abduction, and (**F**) position for hip adduction.

The first position, assessing shoulder adduction, involved gripping the pole close to the armpit area, with participants tightly holding onto the pole using both hands. They also maintained slight contact with the floor by raising up onto their toes with ankles plantar flexed. The knees and hips were flexed and the examined humerus was parallel with the floor whereas the dynamometer was placed in the inner side, fixed on the pole in a position that was adjusted to each subject (Figure 3D). The shoulder abduction was assessed using a similar position to the adduction, but the dynamometer was placed on the outer side of the humerus (Figure 3E). The third position involving the hip adductors was performed in a position where participants had the pole between their legs as close as possible to the pubic symphysis, with the limb to be assessed in a cross-legged position on the top of the other, attempting to squeeze as hard as possible. The supporting leg was on the toes with both knees and hips flexed and the examined femur was parallel with the floor (Figure 3F). Markers (Figure 3A–C) were placed to indicate where the upper arm or lower leg made firm contact with the pole for each position. The dynamometer was then positioned accordingly on the pole for measurement. The distances between the acromion or the ASIS and the center of pressure, as well as the height at which the dynamometer was stabilized on the pole for each position, were recorded for each subject during the re-test.

Participants were instructed to position themselves on the pole, and a submaximal warm-up effort was introduced before the formal testing began. This warm-up phase was not included in data analysis and served solely for familiarization purposes. Each participant was given a standardized command: “Please place the marked portion of your …… (humerus or femur) on the dynamometer”. Grip the pole with your hands and step on your toes as if you are trying to lift your body off the floor. Then squeeze the dynamometer as hard as you can”. Verbal encouragements were given during measurements. The procedure consisted of three maximal strength efforts lasting 5 s each, with a 2-min break in between, for each position and side. The protocol employed isometric ‘make’ tests in compression mode. The same procedure was performed with a 5–7-day interval between sessions for inter-session reliability. Participants were instructed to maintain consistent daily routines and exercise programs on the days of measurement, as well as the preceding and following days. Measurements were taken at the same time each day to avoid circadian effects.

### 2.3. Statistical Analysis

The reliability analysis (intra-session and inter-session) was performed using the intra-class correlation coefficient (ICC1,1), the standard error of measurement (SEM, which is the square root of the within-subject mean squared error from the repeated-measures analysis of variance), the smallest detectable difference (SDD, which is 1.96 × √2 × SEM, expressed as a percentage of the parameter’s grand mean) and the Cronbach’s Alpha [20]. Strength of the reliability was categorized as poor (ICC < 0.40), fair (0.40–0.7), good (0.7–0.9), or excellent (0.9–1.0) [21]. Cronbach’s Alpha (measure of the internal consistency) values were also estimated. The analysis was performed for all the efforts, but also with the first effort excluded (in order to examine if a full effort was needed before the actual measurements familiarization effort). Known-group validity was determined by examining MVIC values in two subgroups based on levels of expertise (professionals and amateurs). It was expected that professionals would have significantly higher values compared to amateur athletes. The Statistical Package for Social Sciences (SPSS, SPSS Inc., Chicago, IL, USA) version 29.0 was used. Confidence intervals were set at 95%. Paired samples t-tests were used to compare the dynamometer MVIC readings between the two limbs. Comparisons were made between left and right limbs or dominant and non-dominant limbs based on WFQ-R results (scores between −7 and −20 as left-footed, -6 and +6 as mixed-footed, +7 and +20 as right-footed) [19] (*p* ≤ 0.05).

The data of the MVIC were measured with a hand-held dynamometer in kilograms (kg). To derive a common metric for reference, the MVIC result in kilograms was transformed to peak torque (in Newton meters) by converting to Newtons (multiplying by 9.80665) and multiplying by the length (in meters) of the lever arm (i.e., the perpendicular distance between the placement of the HHD and the axis of rotation of the tested segment). The lever arm for shoulder adduction was the distance from the acromion to the center of pressure of the dynamometer on the inner side of the humerus (Figure 3A), for shoulder abduction, the distance from the acromion to the center of pressure of the dynamometer on the outer side of the humerus (Figure 3B) and for hip adduction, the distance from the ASIS to the center of pressure of the dynamometer on the inner side of the femur (Figure 3C).

## 3. Results

### 3.1. Participants

Table 1 describes the participants’ characteristics. All participants were female athletes. The mean age and body mass index for this cohort was 29.4 ± 5.9 years and 21.4 ± 1.3 kg/m^2^, respectively. The participants had more than five years of experience, and 65.6% were amateur and 34.4% were professional athletes. Professional athletes had 8.1 ± 2.02 average years of practice and 13.82 ± 6.4 h per week of PD practice, whereas amateur athletes had 3.4 ± 1.8 years of practice and 4.1 ± 2.9 h per week of PD practice respectively (both were found statistically higher in professionals, *p* = 0.000).

Most participants were involved in sport and exotic PD (56.3%), whereas 43.8% in sport. Among the participants, 17 (53.2%) were identified as right-footed, while 15 (46.8%) were categorized as mixed-footed. In this study, none of the subjects experienced joint pain or other orthopedic problems and symptoms.

### 3.2. Intra-Session Reliability

All reliability indices for the intra-session reliability study are in Table 2. The ICCs between the three efforts among all subjects for both days ranged between 0.83 and 0.96 for both body sides, in all the different movements. When the first effort values were excluded, the ICCs exhibited slightly better scores, ranging from 0.87 to 0.96. Similarly, Cronbach’s Alpha values were high. The standard error of measurement (SEM) ranged from 2.24 to 5.02 kg for shoulder adduction, shoulder abduction and hip adduction for both sides for all three efforts and 2.29–4.06 kg for the second and third efforts only. Higher SEM were presented in left hip adduction during both first and second days. The SDD indices varied from 15.08% to 28.92% for all movements for both sides between the three efforts on both days. When the first effort was excluded, the SDD estimates were slightly lower (14.92%%-27.46%). Right hip adduction had the lowest SDD value (14.92%) and left hip adduction had the highest SDD value (27.46%).

### 3.3. Inter-Session Reliability

All data from the inter-session study are summarized in Table 3. The two last efforts were used for estimating the average values. The correlation between the measurements taken on the two days was very high (ICC1,1 range, 0.93–0.97) for all three movements and limb sides. Similarly, Cronbach’s Alpha values were high. The SEM ranged from 1.97 to 2.88 Kg in shoulder adduction and shoulder abduction, whereas the SEM in hip adduction ranged from 3.59 kg (right hip) to 3.72 kg (left hip). The SDD ranged from 15.19% to 22.8% for all three movements and limb sides.

### 3.4. MVIC Values

In Table 4, all different metric values of all movements and sides are presented. There was no statistically significant difference observed in the average MVIC values between the left and right sides for shoulder abduction and hip adduction. However, this was in contrast with shoulder adduction (Table 4). Considering lower limbs, no differences were found between dominant and non-dominant sides in right-footed participants (*p* = 0.532) or mixed-footed participants (*p* = 0.232). Statistically significant differences were found between shoulder abduction and adduction in both upper limbs, with abduction demonstrating higher MVIC values than adduction (*p* = 0.000).

The participants were divided into two groups for subgroup analysis: a professional group (N = 11), and an amateur group (N = 21), based on their level of expertise. In Table 5, MVIC values of all movements and sides are presented for both groups separately. In all movements and sides, professionals had statistically significant higher MVIC values than amateurs. Furthermore, considering all participants, there was a statistically significant moderate to good correlation observed between MVIC values and years of practice (r = 0.516–0.744, *p* < 0.001) as well as between MVIC values and weekly hours of practice (r = 0.398–0.652, *p* < 0.05).

## 4. Discussion

This study represents the first attempt to develop and test a pole dancing-specific muscle strength protocol utilizing a handheld dynamometer fixed on the pole. The protocol developed in this study yielded reproducible intra-session and inter-session measurements when conducted by the same examiner. In the intra-session study, the ICC values for all movements in both limbs were very good to excellent (>0.84), while measurements exhibited a relatively low measurement error (<5 kg), indicating minimal variation among subjects. The SDD was less than 28%, a relatively high value indicating the percentage change necessary to demonstrate a positive impact of any intervention. In terms of inter-session reliability, marginally better results were obtained (ICC > 0.93, SEM < 3.7 kg, SDD < 22.8%) indicating that the proposed protocol can be used reliably within and between different days.

Excluding the first effort from the analysis led to improvements in all reliability estimates. Practice or familiarization efforts have been used by several investigators [22,23] in order to produce more reliable and accurate isometric measurements using hand-held dynamometers. During strength measurements, familiarization should be incorporated as part of the routine process in order to enhance confidence and facilitate tissue compliance around the area of interest [24]. In the current study, when the first effort values were excluded, the ICC, SEM, and SDD values achieved slightly better results, although without having a great impact. Because PD athletes are accustomed to the functional testing conditions employed in the present study through their regular use in training contexts, it is possible that habituation exists, thereby contributing to the maintenance of a high degree of reliability. The utilization of functional MVIC testing positions appears to offer additional advantages compared to other unfamiliar testing conditions, such as enhanced repeatability of measurements.

Comparing the intra-session reliability indexes among different movements across both days (Table 2), the results showed generally a mixed picture with shoulder adduction yielding the best ICC, SEM, and SDD values followed by shoulder abduction. Hip adduction and especially the left hip adduction had the lowest values on the first day but was considerably improved on the second day. As the body position during hip adduction was more unsteady in comparison with shoulder testing positions, one of the main reasons for these results may be the stabilization. It is possible that the unstable reliability values of left hip adduction were due to the fact that it was the non-dominant leg for most of the participants, which might have made it more difficult to maintain it in a correct position and press the HHD. Finally, another explanation might be the high variance between the peak hip values in the subjects, which was not too evident for shoulder measurements. Almost all intra-session indexes were improved on the second day measurements that could also indicate a learning or familiarization effect. Regarding inter-session reliability indexes, a similar picture was evident although the mean strength values from the last two efforts were calculated for each day.

As there are no similar studies in PD, it is not possible to compare the results directly. However, the findings of this study appear to be consistent with those from other studies that assessed the reliability of hand-held dynamometers. Regarding intra-rater reliability, ICC values for shoulder abduction ranged from 0.87 to 0.98 [25] or 0.83 to 0.85 [26] and the minimal detectable change (MDC) ranged from 15% to 35%, whereas ICC values for shoulder adduction ranged from 0.77 to 0.98 and an MDC of 51% [25]. Similarly, for hip adduction and abduction, ICC values ranged from 0.94 to 0.95 [27], or 0.97 to 0.98 [28], or 0.63 to 0.89 [26], and the SEM ranged from 2.3 to 6.8 [27]. Despite the fact that the positions used in the current study were functional, influenced by gravity, and without stabilization, in contrast to previous studies, the results of this study are comparable or in some cases, even better. This can be explained by the fact that the HHD was fixed on the pole rather than being held by the examiner. As the subject strength increases, HHD reliability seemed to decrease, particularly with movements that produce high MVIC values, such as abduction in the plane of the scapula [29], and it was influenced by the rater’s characteristics [30,31].

To the best of our knowledge, relatively few studies have used functional protocols to measure MVIC in athletes. Sport-specific strength protocols have only been developed in two recent studies, one for rock climbing [15] and one for ballet dance [16]. A low-resource maximal isometric finger strength testing protocol was created by Torr et al. [15] using a pulley system to add or remove weight from a climbers’ body, and its test-retest reliability and criterion validity were evaluated. In all cases, the inter-session ICC values were excellent (ICC > 0.91), with low biases and effect sizes, especially when expressed as a percentage of body mass. A portable, barre-mounted external stabilizing dynamometer was employed by Strzelinski et al. [16] to assess hip and lower extremity muscle performance. There was moderate to high inter-rater reliability for all positions, ranging from 0.527 to 0.851. Compared to a static general MVIC testing protocol, a sport-specific testing protocol offers several advantages: (a) position specificity during testing in movements that require extensive range of motion, or gravity influence or multijoint stability; (b) position testing familiarization and optimal reproducibility; and (c) relevance in prescribing load intensities for exercises to improve strength or fatigue.

### 4.1. MVIC Values

The strength values reported in the literature for HHD are reported in force units (kg or N) rather than torque units (Newton-meters), so that comparison purposes cannot be achieved, which is the primary reason for setting reference values [32]. Despite the limited sample size, anthropometric characteristics of participants (such as lever arm length or body mass) were taken into account, and MVICs were expressed in a range of common metric units. MVIC values were not found to differ between the left and right sides or between the dominant and non-dominant sides in shoulder abduction and hip adduction contrary to shoulder adduction. There may be an explanation for this in terms of PD biomechanics. To execute special figures, it is essential to have a strong dominant hand grip to maintain control over the pole, along with strong shoulder adduction to firmly grip the pole for swirling movements on the non-dominant side. Previous studies have demonstrated that the dominant hand has significantly higher hand grip values than the non-dominant [1].

MVIC values were higher in shoulder abduction in comparison to adduction in both upper limbs, although there are no comparable studies. The abductors were significantly (*p* < 0.05) weaker than the adductors in elite junior tennis players in horizontal isokinetic evaluation [33]. These contradictory results, however, could be attributed to differences between sports (tennis being a rather asymmetrical sport), testing protocols, and the age of the participants.

In the current study, known-group validity, a type of construct validity, was determined. As anticipated, professionals were expected to exhibit significantly higher MVIC values compared to amateur athletes. Indeed, the results confirmed this expectation, with professionals demonstrating statistically significant higher MVIC values than amateurs. Furthermore, a statistically significant moderate to good correlation was observed between MVIC values and both years or weekly hours of practice. These findings align with previous studies indicating that handgrip strength in PD women increases progressively with the level of experience [3,34,35].

### 4.2. Protocol Implementation Recommendations

For the protocol to be implemented reliably, certain conditions must be met. To identify potential problems and become familiar with the procedure and pressure pattern, a maximal force test should be performed prior to each measurement for each position and each side. As a result of the round shape of the pole and the shiny construction material, the dynamometer must be very well fixed on the pole in order for the subject to apply a vertical force. Additionally, if the dynamometer causes soreness to the participant, an intermediate cloth material should be placed between the dynamometer and the participant’s skin. Due to the complexity of the positions, it is recommended to present the positions with photographs and hands-on demonstrations when explaining the procedure. It is necessary to record the exact points on the dynamometer where the body applied force in order to standardize the process.

The height at which the dynamometer is stabilized on the pole should also be recorded for every position. In the initial application of each evaluation position, the dynamometer was consistently placed on the pole at the same height for both sides of the body in the present study. For each participant, the contact point between the body part and the dynamometer was fixed at a position that was comfortable for them. It is considered necessary to have stable conditions of temperature and humidity in the surrounding area during the application of the protocol, and the participant should have the option of using magnesia or similar materials to prevent slipping due to perspiration at the points of contact with the pole, whether or not they are examination points. On the days of the measurements as well as the day before and the day after, participants were asked to follow the same daily and exercise program. Both days, the measurements were conducted at the same time.

### 4.3. Clinical Implications

Establishing valid and reliable functional measures of muscle function will enable trainers to prescribe appropriate load intensities for exercises aimed at improving strength or managing fatigue, while also allowing clinicians to monitor the effects of interventions over time within groups of patients. It will also permit comparison of between-group differences (i.e., healthy vs. pathologic conditions). In most HHD protocols, muscle strength evaluations are typically conducted in gravity-neutralized positions for all tested muscle groups, with the participant assuming a fixed, unrealistic position. Rarely do studies utilize evaluations against gravity in a functional position that mimics the demands of the sport, as seen in the protocol of the current study.

### 4.4. Limitations

The conclusions drawn from this study should be considered with awareness of certain limitations. The study’s focus on assessing shoulder/hip strength while neglecting to measure other muscles’ strength presents a notable limitation. Although the study aimed to develop and test a muscle strength protocol specific to PD, the choice of muscles/motions assessed may not fully encompass the strength requirements of a PD athlete. While the protocol demonstrated good reliability, it is important to acknowledge that the selected muscles and motions may not represent the complete spectrum of strength needed for PD. Therefore, future research could benefit from incorporating additional assessments to provide a more comprehensive understanding of PD athletes’ strength profiles. Moreover, dominance of the upper limbs was selected based on writing preference. Future studies may also use ball throwing preference for similar study protocols.

## 5. Conclusions

The results of this study suggest that the strength assessment protocol in sport-specific positions for female PD athletes is a reliable and valid measure suitable for application in both training and clinical practice. It is an easy-to-use protocol for both the athlete and the rater, while also being cost-effective, as it only requires a handheld dynamometer rather than a custom-made specialized device. Nevertheless, the study should be extended to a larger sample, including other positions and muscle groups as well as other categories of athletes (e.g., men, children, and teenagers).

## Figures and Tables

**Table 1:** Sample characteristics.

Parameter	Values (Mean ± SD)
Age	29.37 ± 5.88 yrs
Weight	57.92 ± 5.45 kg
Height	164.09 ± 5.13 cm
BMI	21.41 ± 1.34 kg/m^2^
Level of expertise	N = 21 (65.6%) amateur, N = 11 (34.4%) professional
Total years of practice	5.02 ± 2.92
Hours per week of PD practice	7.44 ± 6.38
Hours per week of other sports practice	2.37 ± 4.02
WFQ-R_m_	4.69 ± 3.19
WFQ-R_s_	2.34 ± 4.00
WFQ-R_t_	7.09 ± 5.77
	Values (Frequency and percent)
Type of training	N = 18 (56.3%) sport+ exotic N = 14 (43.8%) sport
Other sports	N = 18 (56.3%) NO N = 14 (43.8%) YES
Dominant upper limb	N = 29 (91%) Right N = 3 (9%) Left
Dominant lower limb	N = 17 (53.2%) Right, Ν = 0 (0.0%) Left, N = 15 (46.8%) mixed

Abbreviations: BMI = Body Mass Index; WFQ-Rm = mobility score of Waterloo Footedness Questionnaire- Revised, WFQ-Rs = stability score of Waterloo Footedness Questionnaire- Revised, WFQ-Rt = total score of Waterloo Footedness Questionnaire- Revised.

**Table 2:** Intra-session reliability for the first and second day of measurements including 3 efforts or 2 last efforts.

Intra-Session (*n* = 32)	MVIC (Kg) Mean (SD)			Reliability (3 Efforts)				Reliability (2 Last Efforts)			
	*1 Effort*	*2 Effort*	*3 Effort*	*ICC (95% CI)*	*SEM*	*SDD*	*Cronbach’s* *Alpha*	*ICC (95% CI)*	*SEM*	*SDD*	*Cronbach’s* *Alpha*
Day 1 (test)											
Shoulder adduction Left Right	34.73 (7.12) 33.73 (8.69)	35.85 (8.29) 34.33 (9.49)	36.39 (7.44) 34.16 (8.53)	0.865 (0.775–0.926) 0.923 (0.867–0.959)	2.72 2.50	21.14 20.34	0.954 0.972	0.903 (0.813–0.952) 0.922 (0.848–0.961)	2.45 2.55	18.80 20.64	0.949 0.958
Shoulder abduction Left Right	35.76 (7.96) 36.21 (8.23)	36.43 (7.51) 37.37 (8.23)	36.56 (7.76) 38.21 (7.75)	0.865 (0.774–0.926) 0.862 (0.769–0.924)	2.86 2.89	21.87 21.50	0.950 0.953	0.898 (0.804–0.949) 0.842 (0.702–0.919)	2.47 3.17	18.76 23.25	0.945 0.915
Hip adduction Left Right	54.88 (13.37) 56.01 (14.73)	58.19 (16.81) 56.21 (13.87)	59.71 (16.26) 56.76(12.72)	0.833 (0.724–0.908) 0.890 (0.814–0.940)	6.01 4.63	28.92 22.78	0.945 0.959	0.875 (0.762–0.935) 0.890 (0.789–0.945)	5.84 4.46	27.46 21.88	0.934 0.940
Day 2 (re-test)											
Shoulder adduction Left Right	34.88 (6.16) 34.31 (7.72)	35.66 (7.02) 35.59 (7.21)	36.27 (6.53) 35.78 (8.14)	0.865 (0.774–0.926) 0.870 (0.782–0.929)	2.36 2.71	18.37 21.32	0.953 0.955	0.885 (0.780–0.942) 0.918 (0.848–0.960)	2.29 3.04	17.65 23.61	0.940 0.915
Shoulder abduction Left Right	36.74 (8.02) 37.95 (8.40)	37.39 (7.95) 38.71 (9.03)	37.85 (9.29) 39.10 (9.23)	0.927 (0.874–0.961) 0.925 (0.871–0.960)	2.24 2.41	16.63 17.31	0.975 0.974	0.924 (0.851–0.962) 0.929 (0.861–0.965)	2.38 2.45	17.53 17.45	0.960 0.962
Hip adduction Left Right	57.42 (14.78) 57.80 (15.04)	59.22 (15.11) 58.80 (16.37)	58.72 (13.63) 58.24 (15.05)	0.880 (0.798–0.935) 0.958 (0.927–0.978)	5.02 3.17	23.80 15.08	0.957 0.986	0.922 (0.848–0.961) 0.960 (0.921–0.980)	4.06 3.15	19.08 14.92	0.958 0.980

Abbreviations: Kg = Kilograms, SD = Standard Deviation.

**Table 3:** Inter-session reliability including the average values of the last 2 efforts.

Inter-Session (*n* = 32)	MVIC (kg) Mean (SD)		Reliability			
Average	Day 1	Day 2	ICC (95% CI)	SEM	SDD	Cronbach’s Alpha
Shoulder adduction Left Right	36.19 (7.68) 34.25 (8.83)	35.96 (6.58) 35.38 (7.38)	0.962 (0.922–0.981) 0.927 (0.851–0.964)	1.97 2.88	15.19 22.86	0.960 0.933
Shoulder abduction Left Right	36.49 (7.43) 37.79 (7.67)	37.62 (8.48) 38.90 (8.96)	0.936 (0.869–0.968) 0.944 (0.885–0.972)	2.71 2.66	20.24 19.19	0.939 0.947
Hip adduction Left Right	58.95 (16.01) 56.49 (12.93)	58.97 (14.10) 58.52 (15.56)	0.970 (0.938–0.985) 0.963 (0.925–0.982)	3.72 3.59	17.49 17.32	0.969 0.967

Abbreviations: Kg = Kilograms, SD = Standard Deviation.

**Table 4:** MVIC in different average metric values (mean values and standard deviations) and comparisons between sides.

	kg Aveg	kg Max	N Aveg	*p* Value	Νm Aveg	*p* Value	Νm/kg Aveg	*p* Value
Shoulder adduction Left Right	36.19 (7.68) 34.25 (8.83)	38.17 (7.69) 36.21 (8.89)	354.20 (75.32) 355.85 (86.67)	0.034	20.93 (3.99) 13.89 (3.92)	0.000	0.36 (0.07) 0.24 (0.07)	<0.001
Shoulder abduction Left Right	36.49 (7.43) 37.79 (7.67)	38.35 (7.67) 39.69 (8.39)	357.89 (72.90) 370.58 (75.25)	0.131	33.52 (5.88) 34.80 (6.60)	0.105	0.58 (0.10) 0.60 (0.12)	0.086
Hip adduction Left Right	58.95 (16.01) 56.49 (12.93)	62.71 (16.46) 59.78 (14.23)	578.10 (157.04) 553.98 (126.77)	0.243	23.89 (6.98) 22.95 (5.99)	0.282	0.42 (0.13) 0.40 (0.12)	0.340

Abbreviations: aveg = average, max = maximum, Kg = kilogram, N = Newton, Nm = Newton meter.

**Table 5:** MVIC values (mean values and standard deviations) in professional and amateur PD athletes.

	Nm/kg (Total) N = 32	Nm/kg (Professionals) N = 11	Nm/kg (Amateurs) N = 21	*p* Value
Shoulder adduction Left Right	0.36 (0.07) 0.24 (0.07)	0.41 (0.05) 0.32 (0.05)	0.34 (0.07) 0.20 (0.04)	0.003 * 0.000 *
Shoulder abduction Left Right	0.58 (0.10) 0.60 (0.12)	0.62 (0.12) 0.68 (0.15)	0.56 (0.09) 0.60 (0.09)	0.012 * 0.036 *
Hip adduction Left Right	0.42 (0.13) 0.40 (0.12)	0.54 (0.11) 0.52 (0.11)	0.35 (0.08) 0.34 (0.06)	0.000 * 0.000 *

*: Indicates statistically significant difference in MVIC values. Abbreviations: Kg = kilogram, Nm = Newton meter.

## Data Availability

Data are available upon reasonable request from the corresponding author.
